# Retrospective analysis of ustekinumab in Crohn’s disease between biologic-naïve and biologic-experienced

**DOI:** 10.3389/fgstr.2026.1926425

**Published:** 2026-09-22

**Authors:** Kaifei Yuan, Xianhong Hu, Qi Fang, Siyuan Li, Ping Lin, Baijing Ding, Tongyuan Lin

**Affiliations:** 1 Postgraduate School, Anhui Normal University, Wuhu, China; 2 Postgraduate School, Wannan Medical University, Wuhu, China; 3 The Department of Gastroenterology, The First People’s Hospital of Wu Hu, Wuhu, China

**Keywords:** clinical efficacy, Crohn’s disease, psychological status, quality of life, ustekinumab

## Abstract

**Objective:**

This thesis investigates the differences in clinical efficacy of ustekinumab (UST) as a biologic-naïve or biologic-experienced for Crohn’s Disease (CD) patients in terms of clinical manifestations and laboratory test indicators. It also explores the correlation between clinical response status and patients’ quality of life, as well as their anxiety and depression levels.

**Method:**

This study involved 61 CD patients treated with UST. We gathered clinical data, laboratory results, CDAI scores and SES-CD scores at 0, 8, and 32 week, and assessed quality of life and psychological state at baseline and week 32. Initially, patients were divided into biologic-naïve and biologic-experienced groups based on their previous biologic history. By analyzing the changes in laboratory indicators at weeks 0, 8, and 32, as well as the differences in the degree of these changes, the effectiveness of different treatment regimens was evaluated. At the end of the treatment (week 32), 61 patients were reclassified into the clinical response group and the non-response group based on the decrease in CDAI score. Subsequently, statistical methods were used to assess the differences in quality of life and psychological status between these two response groups.

**Results:**

There was no statistically significant difference in the baseline data between the biologic-naïve and biologic-experienced groups (*P* > 0.05). After treatment, the inflammatory and nutritional indicators in both groups improved. The inflammation decreased more significantly in the biologic-naïve group. At 32 week, the reduction in CDAI and SES-CD in the biologic-naïve group was better than that in the treated group (all *P* = 0.03), but there was no difference in clinical response and remission rate between the two groups. Regardless of whether there was clinical response, the IBDQ score of patients increased compared to the baseline. The improvement in IBDQ in the clinical response group was higher than that in the clinical non-response group (*P* = 0.001). Only the anxiety subscale (HADS-A) of the response group, significantly decreased (*P* = 0.02), while the depression score of HADS-D showed no significant improvement. There were no statistically significant differences in the anxiety subscale (HADS-A) and depression subscale (HADS-D) of the rnon-esponse group (*P* > 0.05).

**Conclusion:**

UST can improve the inflammation and nutritional status of patients with CD who are biologic-naïve or biologic-experienced. The clinical and endoscopic improvement benefits for the biologic-naïve are more prominent. The quality of life of patients who achieved a clinical response has improved more significantly. Anxiety symptoms can be somewhat alleviated, while the depressive state has not shown any significant change.

## 1 Introduction

Inflammatory bowel disease (IBD) is a gastrointestinal disorder characterized by chronic inflammation that can result in irreversible damage, marked by prolonged duration and frequent relapses (1). Recent epidemiological data indicate a rising incidence of IBD in Asia, while the incidence rate in North America and Europe remains at approximately 30% (2, 3). Over the past three decades, both the prevalence and incidence of IBD in China have markedly increased, with current prevalence rates ranging from 60 to 70 per 100,000 individuals. Projections suggest that the burden of IBD in China will continue to escalate over the next 25 years, posing a significant public health challenge (4). IBD encompasses two primary forms: Crohn’s disease (CD) and ulcerative colitis (UC). CD can affect the entire gastrointestinal tract, from the oral cavity to the anus, manifesting as a chronic condition with recurrent episodes and variable severity (1). In the absence of timely and effective interventions, CD may progress to severe complications such as intestinal strictures and perforations, thereby significantly diminishing patients’ quality of life.

The primary therapeutic approaches for CD currently encompass pharmacological treatment and surgical intervention, with pharmacotherapy serving as the foundational element. Clinical investigations have demonstrated that biologic agents exhibit significantly greater efficacy compared to traditional medications in the management of CD. Notably, ustekinumab (UST) has been recognized as a first-line biologic agent recommended for the induction and maintenance of remission in moderate to severe cases (5). UST is a human immunoglobulin G monoclonal antibody that selectively binds to interleukin (IL)-12/23, functioning as a direct receptor antagonist (6). It specifically targets the IL-23p40 subunit, thereby inhibiting the binding of IL-12/23 to the IL-12Rβ1 receptor, which disrupts signal transduction pathways involved in inflammation. This action inhibits the activation of downstream immune cells, such as natural killer (NK) cells and T cells, reduces the secretion of inflammatory mediators by effector cells, and consequently mitigates chronic intestinal inflammation (7). UST has been shown to effectively induce and maintain clinical remission, promote endoscopic mucosal healing, and enhance patients’ nutritional status (8).

The pathogenesis of CD remains inadequately understood, and its chronic nature profoundly impacts patients’ psychological well-being, frequently resulting in anxiety and depression. These psychological conditions can exacerbate intestinal inflammation through neuroendocrine-immune pathways, including the hypothalamic-pituitary-adrenal axis and the autonomic nervous system, which release inflammatory mediators and perpetuate a detrimental cycle of bidirectional brain-gut interaction (9, 10). The “2023 China Crohn’s Disease Diagnosis and Treatment Guidelines (Guangzhou Edition)” underscore that the therapeutic objective for CD patients should encompass achieving clinical remission, improving serum and fecal inflammatory markers, and enhancing quality of life (11). In contrast to previous guidelines, this edition not only underscores symptom management but also places increased emphasis on the psychological health and overall living conditions of patients. Consequently, the management of CD should not only target the control of intestinal inflammation but also address patients’ mental health and quality of life, striving for comprehensive, multidimensional care (12–14). While existing studies have established the safety and efficacy of UST in the treatment of CD, there remains a relative paucity of research concerning its effects on patients’ psychological changes and quality of life.

This study undertook a retrospective analysis of 61 CD patients who received UST treatment in the Gastroenterology Department of Wuhu First People’s Hospital (hereafter referred to as “our hospital”) from April 2022 to May 2025. The analysis rigorously compared the clinical outcomes of UST as biologic-naïve versus biologic-experienced, alongside evaluating differences in quality of life and psychological state between the clinical response group and the non-response group. The findings aim to provide a multi-dimensional foundation for optimizing clinical UST treatment strategies, facilitating individualized assessment of therapeutic outcomes, and enhancing the comprehensive physical and mental health management of CD patients throughout their treatment cycle.

## 2 Materials and methods

### 2.1 Study subjects

Sixty-one patients with CD who received UST treatment in the gastroenterology department of our hospital between April 2022 and May 2025 were selected based on stringent inclusion and exclusion criteria. These patients were categorized into two groups based on their history of biological treatment failure: the biologic-naïve group (n=21) and the biologic-experienced group (n=40). The biologic-naïve group comprised CD patients who had not previously received biological treatment, with UST being their initial biological therapy. In contrast, the biologic-experienced group consisted of CD patients who had previously received treatment with one biologic agent and failed, then switched to UST therapy.The initiation of UST treatment was designated as the baseline (week 0), with the primary endpoint set at Week 32 and the secondary endpoint at Week 8. All patients underwent evaluations of their quality of life and psychological state at week 0 of the treatment. Comprehensive clinical data for patients treated with UST were systematically gathered via the hospital information system. This included demographic information (gender, age, disease duration), Montreal classification (details refer to **Supplementary Appendix 1**), and previous biological treatments. Additionally, laboratory indicators such as C-reactive protein (CRP), erythrocyte sedimentation rate (ESR), hemoglobin (Hb) and albumin (ALB) were documented for each instance of medication administration. Furthermore, the patients’ disease activity index and endoscopic scores were recorded. These data were utilized to assess the differences in clinical efficacy of UST when used as biologic-naïve versus biologic-experienced.

At the end of the treatment (at 32 week), based on the decline in the CDAI score, we reclassified 61 patients into the clinical response group (n=34) and the clinical non-response group (n=27). Among them, the response group consisted of 13 patients from the biologic-naïve and 21 patients from the biologic-experienced, while the non-response group comprised 8 patients from the biologic-naïve and 19 patients from the biologic-experienced. We then re-evaluated the psychological state and quality of life of the two groups of patients after the end of treatment, and compared the differences between the baseline and the end-point of each group, as well as the differences in the degree of change in psychological state and quality of life between the response group and the non-response group. This study employed a single-center retrospective cohort design, aiming to compare and analyze the differences in quality of life and psychological state between the clinical response group and the non-response group. This study received approval from the hospital’s ethics committee (Ethics Number: YYLL20240065).

### 2.2 Inclusion and exclusion criteria

#### 2.2.1 Inclusion criteria

1. Patients diagnosed with Crohn’s Disease (CD) in accordance with the diagnostic criteria outlined in the “Chinese Guidelines for the Diagnosis and Treatment of Crohn’s Disease (2023, Guangzhou)” are selected.
2. Patients must undergo regular Ustekinumab (UST) treatment for a minimum period of 32 week.
3. Patients are required to provide blood samples at baseline (0 weeks), 8 week, and 32 week, and must complete laboratory evaluations including a complete blood cell count, comprehensive biochemical analysis, and assessment of inflammatory markers.
4. Participants must have provided informed consent for participation in this research study, willingly engaged, and furnished accurate and valid information.
5. Participants are expected to complete the relevant scale assessment at the baseline (0 weeks) and at the conclusion of the 32-week treatment period.

#### 2.2.2 Exclusion criteria

1. Individuals with a history of smoking, alcohol abuse, impaired coagulation function, other active autoimmune diseases, malignant tumors, or chronic conditions such as hypertension, diabetes, chronic liver disease, or chronic kidney disease;
2. Patients who decline UST treatment;
3. Individuals unable to comprehend or complete the questionnaire;
4. Recent systemic glucocorticoid exposure within 12 weeks prior to the initiation of biological agents;
5. Individuals diagnosed with mental disorders who are currently prescribed antipsychotic medications.

### 2.3 Administration method and timing

The administration of medication for patients is conducted in accordance with the “ECCO Guidelines on Therapeutics in Crohn’s Disease: Medical Treatment” (5). The UST administration protocol is delineated as follows: patients with a body weight of ≤55 kg receive an intravenous dose of UST 260 mg; those with a body weight between 55 kg and 85 kg receive an intravenous dose of UST 390 mg; and patients with a body weight exceeding 85 kg receive an intravenous dose of UST 520 mg. Subsequently, a subcutaneous injection of UST 90 mg is administered every 8 week. The study commences at the initial administration (week 0) of UST for patients with CD and continues with administration every 8 week. The study endpoint is established at 32 week following the initiation of medication.

### 2.4 Disease assessment

#### 2.4.1 Clinical efficacy evaluation

This study employed the Crohn’s Disease Activity Index (CDAI) (refer to **Supplementary Appendix 2**) to evaluate disease efficacy, specifically focusing on clinical response and clinical remission. Clinical response is defined as a decrease of ≥100 points in the CDAI score after treatment compared to the baseline, while clinical remission is defined as a CDAI score of <150 points.

#### 2.4.2 Endoscopic efficacy evaluation

In this study, the efficacy of treatment was assessed using the Simplified Endoscopic Crohn’s Disease Activity Score (SES-CD). Patients with CD underwent colonoscopic evaluation both at baseline and after 32 week of treatment. Two gastroenterologists, ZY and LP, who were blinded to the patients’ clinical data, independently applied the SES-CD scoring system. This system evaluates four intestinal segments—the terminal ileum, ascending colon, transverse colon, and descending colon/rectum—across four dimensions: ulcer area, ulcer number, severity of mucosal inflammation, and lesion severity. The cumulative score ranges from 0 to 16. Core response criteria: The total SES-CD score decreased by≥50% compared to baseline, and no new intestinal segments were affected. Endoscopic remission criteria: The SES-CD total score ≤ 2, individual scores for each intestinal segment ≤ 1, and no active lesions such as ulcers, stenosis, etc. Mucosal healing criteria: Mucosal repair can be observed under endoscopy, without ulcers, erosion, congestion, edema.

#### 2.4.3 Quality of life assessment and psychological state scales

The Inflammatory Bowel Disease Questionnaire (IBDQ) comprises four dimensions: intestinal symptoms, systemic symptoms, emotional function, and social function, encompassing a total of 32 items. Each item is rated on a scale from 1 to 7, where 1 signifies the most severe impact and 7 indicates no impact. The cumulative score is obtained by summing the scores of all items, yielding a total score ranging from 32 to 224 (specific items are detailed in **Supplementary Appendix 3**). A higher total score reflects an improved quality of life for the patient. The Hospital Anxiety and Depression Scale (HADS) consists of two sub-scales: the anxiety sub-scale (HADS-A) and the depression sub-scale (HADS-D), each containing 7 items. The scoring system ranges from 0 to 3, where 0 denotes “almost none,” 1 denotes “sometimes,” 2 denotes “most of the time,” and 3 denotes “almost always.” Notably, the 7th item of the Hospital Anxiety and Depression Scale-Anxiety subscale (HADS-A) and the 1st, 3rd, 5th, and 7th items of the Depression subscale (HADS-D) are reverse-scored. The reverse scoring protocol assigns 3 points to “almost none,” 2 points to “sometimes,” 1 point to “most of the time,” and 0 points to “almost always.” The remaining positively scored items are evaluated according to their original response options (specific items are detailed in **Supplementary Appendix 3**). The total scores for each subscale are calculated independently, ranging from 0 to 21 points. A cumulative score of 0 to 7 suggests no significant anxiety or depression, a score of 8 to 10 suggests a possible presence of anxiety or depression, and a score of 11 to 21 indicates a pronounced presence of anxiety or depression.

### 2.5 Statistical analysis

Statistical analyses were conducted utilizing SPSS version 27.0. Categorical variables were reported as frequencies (n) and percentages (%). The 95% confidence interval (95% CI) was calculated using the Wilson score method. Continuous variables were subjected to normality testing; those conforming to a normal distribution were presented as mean ± standard deviation ( $\bar{\text{x}}$ ± s), whereas non-normally distributed variables were described using the median and interquartile range [M (Q1, Q3)]. For intra-group comparisons at weeks 0 and 8, as well as weeks 0 and 32, the choice of statistical method was contingent upon the normality of the data: paired t-tests were employed for data with a normal distribution, while Wilcoxon signed-rank tests with Bonferroni correction were utilized for data that did not follow a normal distribution. Inter-group comparisons were conducted using the baseline difference method, assessing changes from baseline at weeks 8 and 32. Independent samples t-tests were applied to normally distributed data, whereas Mann-Whitney U tests were used to compare differences in change values between groups for data that were not normally distributed. A P-value of less than 0.05 was deemed to indicate statistical significance in this study.

## 3 Results

### 3.1 Basic information of the study population

A total of 61 patients diagnosed with CD were ultimately included in the study. The cohort was divided into two treatment groups: the biologic-naïve group, comprising 21 patients, and the biologic-experienced group, consisting of 40 patients. Of the total participants, 37 were male, representing 60.7% of the sample, while 24 were female, accounting for 39.3%. The age of onset was predominantly observed in type A2 (17–40 years), with 48 cases (78.7%), followed by type A3 (>40 years) with 13 cases (21.3%). Regarding lesion location, 13 cases (21.3%) were classified as type L1 (terminal ileum), 3 cases (4.9%) as type L2 (colon), and 45 cases (73.8%) as type L3 (ileocolonic). The disease behavior was primarily type B1 (non-stenotic, non-perforating), observed in 53 cases (86.9%), with type B2 (stenotic) in 7 cases (11.5%), and type B3 (perforating) in 1 case (1.6%). Additionally, 17 patients (27.9%) presented with perianal lesions, and 3 patients (4.9%) had intestinal fistulas. Detailed data are presented in **Table 1**.

**Table 1:** General conditions of all patients.

Clinical data	Number of cases	Constituent ratio (%)
Gender		
Male	37	60.7%
Female	24	39.3%
Age of onset		
A1:≤16	0	/
A2:17-40	48	78.7%
A3:>40	13	21.3%
Diseased region		
L1:terminal ileum	13	21.3%
L2:colon	3	4.9%
L3:ileocolon	45	73.8%
L4:upper gastrointestinal tract	0	/
Disease behavior		
B1:Non-narrow and non-penetrating type	53	86.9%
B2:constricting type	7	11.5%
B3:penetrating type	1	1.6%
combination of perianal lesions	17	27.9%
union of intestinal fistula	3	4.9%

Further comparative analysis was conducted on the general clinical data of the two groups at the beginning of the study. There were no significant differences between the two groups in terms of gender, age, disease duration, clinical relevant laboratory indicators, CDAI score and SES-CD score. Detailed data are presented in **Table 2**. The clinical characteristics of CD patients in the initial treatment group with biological reagents and the group with biological agent experience were analyzed and compared. According to the Montreal classification, the age at diagnosis was predominantly distributed within stage A2. Within this stage, the initial treatment group receiving biological agents comprised 14 cases (66.7%), while the experienced group included 34 cases (85%). The subsequent stage, A3, consisted of 7 cases (33.3%) in the initial treatment group and 6 cases (15%) in the experienced group. Concerning the lesion site, the initial treatment group exhibited 5 cases (23.8%) of L1 (terminal ileum) and 16 cases (76.2%) of L3 (ileocolon). In contrast, the experienced group presented with 8 cases (20%) of L1 (terminal ileum), 3 cases (7.5%) of L2 (colon), and 29 cases (72.5%) of L3 (ileocolon). There were no patients with L4 (upper gastrointestinal tract) involvement in either group. Regarding disease behavior, the initial treatment group had 17 cases (81%) classified as B1 (non-stenosis, non-perforation), 4 cases (19%) as B2 (stenosis), and 7 cases (33.3%) as P (perianal lesion). The experienced group, on the other hand, comprised 36 cases (90%) of B1 (non-stenosis, non-perforation), 3 cases (7.5%) of B2 (stenosis), 1 case (2.5%) of B3 (perforation), and 13 cases (32.5%) of P (perianal lesion). Detailed data are presented in **Table 3**.

**Table 2:** Baseline data of the two groups of patients.

Clinical data	The biologic-naïve (n=21)	The biologic-experienced (n=40)	*P*
Gender			0.131
Male	10 (47.6%)	27 (67.5%)	
Female	11 (52.8%)	13 (32.5%)	
Age	30.05 ± 7.25	31.98 ± 9.32	0.235
Disease duration	2.7 ± 1.6	4.1 ± 2.3	0.063
Clinical index			
CDAI	254.56 ± 43.26	237.96 ± 59.15	0.680
SES-CD	8.92 ± 1.79	9.11 ± 1.45	0.660
Laboratory index			
CRP (mg/L)	2.81 (1.49, 11.31)	4.33 (2.05, 11.36)	0.569
Hb (g/L)	131.57 ± 20.31	134.76 ± 23.11	0.572
ESR (mm/h)	17 (11.5, 32.5)	18 (7.25, 31.25)	0.627
WBC (×10^9^/L)	5.21 (4.49, 6.87)	5.87 (4.96, 7.21)	0.202
PLT (×10^9^/L)	273.12 ± 73.89	264.70 ± 89.50	0.713
RBC (×10^12^/L)	5.11 ± 0.48	4.77 ± 0.66	0.338
ALB (g/L)	43.94 ± 8.01	43.75 ± 4.12	0.407
Previous biologic agents			
infliximab	/	22 (55%)	/
adalimumab	/	7 (17.5%)	/
vedolizumab	/	11 (27.5%)	/

Categorical variables are reported as frequencies (n) and percentages (100%). independent sample t-tests and Mann-Whitney U test; *P<0.05, **P<0.01, ***P<0.001.

**Table 3:** The Montreal classification of the two groups of patients.

Project	The biologic-naïve (n=21)	The biologic-experienced (n=40)
Diagnosis age		
A1:≤16	0	0
A2:17-40	14 (66.7%)	34 (85%)
A3:>40	7 (33.3%)	6 (15%)
Site of onset		
L1	5 (23.8%)	8 (20%)
L2	0	3 (7.5%)
L3	16 (76.2%)	29 (72.5%)
L4	0	0
Disease behavior		
B1	17 (81%)	36 (90%)
B2	4 (19%)	3 (7.5%)
B3	0	1 (2.5%)
P	7 (33.3%)	13 (32.5%)

Categorical variables are reported as frequencies (n) and percentages (100%).

### 3.2 Clinical indicator evaluation

#### 3.2.1 Intra-group comparison of the biologic-naïve group

##### 3.2.1.1 Comparative of baseline (0 week) and 8 week

The clinical indicators of patients undergoing biologic-naïve were analyzed and compared. The results demonstrated that, compared to baseline measurements at week 0, there was a statistically significant improvement in ESR levels (*P* < 0.05). Although other indicators, such as CRP, WBC, ALB, PLT, RBC, and Hb, also showed improvements, these differences did not reach statistical significance (*P* > 0.05). Comprehensive results are provided in **Table 4**.

**Table 4:** Comparison of clinical indicators between the biologic-naïve group at 0 week and 8 week.

Project/time	0 weeks	8 week	*P*
CRP (mg/L)	2.81 (1.49, 11.31)	2.10 (1.47, 6.18)	0.140
WBC (×10^9^/L)	6.29 ± 2.11	5.94 ± 2.51	0.149
ALB (g/L)	43.94 ± 8.01	45.60 ± 6.95	0.120
PLT (×10^9^/L)	273.12 ± 73.89	259.10 ± 100.13	0.080
RBC (×10^12^/L)	5.11 ± 0.48	4.96 ± 0.78	0.240
Hb (g/L)	131.57 ± 20.31	140.86 ± 29.52	0.170
ESR (mm/h)	17.0 (11.5, 32.5)	11.0 (5.0, 16.0)	0.010*

paired t-tests and Wilcoxon signed-rank test; *P<0.05, **P<0.01, ***P<0.001.

##### 3.2.1.2 Comparison of baseline (0 week) and 32 week

A comparative analysis of clinical indicators was conducted for patients in the biologic-naïve group. The findings indicated that, relative to baseline measurements at 0 weeks, there were significant improvements in the levels of CRP, PLT, Hb and ESR at 32 week of treatment, with statistically significant differences observed (*P* < 0.05). Conversely, no statistically significant differences were noted in WBC, ALB, and RBC (*P* > 0.05), as detailed in **Table 5**.

**Table 5:** Comparison of clinical indicators between the biologic-naïve group at 0 week and 32 week.

Project/time	0 week	32 week	*P*
CRP (mg/L)	2.81 (1.49, 11.31)	1.89 (0.39, 5.91)	0.005**
WBC (×10^9^/L)	6.29 ± 2.11	5.64 ± 1.58	0.896
ALB (g/L)	43.94 ± 8.01	50.14 ± 5.49	0.050
PLT (×10^9^/L)	273.12 ± 73.89	240.81 ± 69.39	0.040*
RBC (×10^12^/L)	5.11 ± 0.48	5.53 ± 1.01	0.080
Hb (g/L)	131.57 ± 20.31	149.29 ± 25.01	0.040*
ESR (mm/h)	17 (11.5, 32.5)	10.0 (5.0, 16.0)	0.003**

paired t-tests and Wilcoxon signed-rank test; *P<0.05, **P<0.01, ***P<0.001.

#### 3.2.2 Intra-group comparison of the biologic-experienced group

##### 3.2.2.1 Comparative of baseline (0 week) and 8 week

A comparative analysis of the clinical indicators in the biologic-experienced group was conducted. The findings indicated a significant reduction in the ESR at 8 week of treatment when compared to baseline measurements (0 weeks), with a statistically significant difference (*P* < 0.05). Other clinical indicators, including PLT, CRP, WBC, ALB, RBC, and Hb, also demonstrated improvement relative to baseline; however, these differences were not statistically significant (*P* > 0.05). For further details, please refer to **Table 6**.

**Table 6:** Comparison of clinical indicators between the biologic-experienced group at 0 week and 8 week.

Project/time	0 week	8 week	*P*
CRP (mg/L)	4.33 (2.05, 11.36)	2.48 (1.61, 9.61)	0.054
WBC (×10^9^/L)	5.81 ± 2.38	7.02 ± 2.55	0.307
ALB (g/L)	43.75 ± 4.12	48.35 ± 3.21	0.120
PLT (×10^9^/L)	264.70 ± 89.5	244.38 ± 54.16	0.080
RBC (×10^12^/L)	4.77 ± 0.66	4.32 ± 1.25	0.060
Hb (g/L)	134.76 ± 23.11	146.83 ± 30.83	0.130
ESR (mm/h)	18 (7.25, 31.25)	9.50 (7.00, 20.50)	0.002**

paired t-tests and Wilcoxon signed-rank test; *P<0.05, **P<0.01, ***P<0.001.

##### 3.2.2.2 Comparison of baseline (0 weeks) and 32 week

The clinical indicators of patients undergoing biologic-experienced were analyzed and compared. The findings revealed that, relative to baseline measurements (week 0), there was a statistically significant improvement in the levels of ALB, PLT, RBC, Hb, and ESR after 32 week of treatment (*P* < 0.05). Conversely, no statistically significant differences were observed in CRP and WBC levels (*P* > 0.05). For further details, refer to **Table 7**.

**Table 7:** Comparison of clinical indicators between the biologic-experienced group at 0 weeks and 32 week.

Project/time	0 week	32 week	*P*
CRP (mg/L)	4.33 (2.05, 11.36)	2.43 (0.74, 8.18)	0.050
WBC (×10^9^/L)	5.81 ± 2.38	7.06 ± 1.68	0.742
ALB (g/L)	43.75 ± 4.12	53.51 ± 6.61	0.010*
PLT (×10^9^/L)	264.70 ± 89.5	225.36 ± 54.33	0.020*
RBC (×10^12^/L)	4.77 ± 0.66	5.37 ± 1.21	0.040*
Hb (g/L)	134.76 ± 23.11	153.16 ± 19.52	0.010*
ESR (mm/h)	18 (7.25, 31.25)	12.00 (6.25, 19.75)	0.001**

paired t-tests and Wilcoxon signed-rank test; *P<0.05, **P<0.01, ***P<0.001.

### 3.3 Comparison of the variation range of clinical indicators

#### 3.3.1 Comparison of the changes in clinical indicators at 8 week

At the 8-week treatment interval, an additional analysis was performed to assess the differences in the variation ranges of laboratory test indicators between the biologic-naïve group and the biologic-experienced group. The findings revealed that the ΔCRP and ΔESR were significantly greater in the naive group compared to the biologic-experienced group (*P* = 0.046, *P* = 0.032), indicating a more pronounced reduction in CRP and ESR levels in the naive group at 8 week. Conversely, the ΔALB and ΔRBC were significantly smaller in the biologic-naïve group than in the biologic-experienced group (*P* = 0.001, *P* = 0.011), suggesting a more substantial increase in albumin and red blood cells in the biologic-experienced group.No statistically significant differences were observed in the ΔWBC, ΔPLT, and ΔHb between the two groups (*P* > 0.05). For further details, refer to **Table 8**.

**Table 8:** Comparison of changes in clinical indicators between the two groups of patients at 8weeks.

Project/group	The biologic-naïve	The biologic-experienced	*P*
ΔCRP	0.53 (-0.34, 4.93)	0.43 (-0.59, 2.47)	0.046*
ΔWBC	0.35 ± 0.40	-1.39 ± 0.17	0.236
ΔALB	-1.66 ± 1.06	-4.6 ± 0.91	0.001**
ΔPLT	14.02 ± 26.24	20.32 ± 35.34	0.433
ΔRBC	0.15 ± 0.30	0.45 ± 0.59	0.011*
ΔHb	-9.29 ± 9.21	-12.07 ± 7.27	0.238
ΔESR	6.0 (2.5, 14.5)	2.00 (-0.75, 12.50)	0.032*

Δ=0 weeks−8 week; independent sample t-tests and Mann-Whitney U test.

*P<0.05, **P<0.01, ***P<0.001.

#### 3.3.2 Comparison of the changes in clinical indicators at 32 week

At 32 week, the ΔCRP and ΔESR values in the biologic-naïve group were greater than those in the treated group (*P* = 0.030, *P* = 0.048). The reduction in inflammatory markers CRP and ESR was more significant in the biologic-naive group. Meanwhile, the ΔALB and ΔPLT values in the biologic-naïve group were lower than those in the biologic-experienced group (all *P* = 0.001), suggesting that the improvement in albumin elevation and platelet decrease in the treated group was better than that in the biologic-naïve group. There were no statistically significant differences in ΔWBC, ΔRBC, and ΔHb between the two groups (*P* > 0.05), as detailed in **Table 9**.

**Table 9:** Comparison of changes in clinical indicators between the two groups of patients at 32 week.

Project/group	The biologic-naïve	The biologic-experienced	*P*
ΔCRP	1.37 (0.59, 7.21)	1.23 (-0.73, 3.79)	0.030*
ΔWBC	0.65 ± 0.53	-1.25 ± 0.70	0.678
ΔALB	-6.2 ± 2.52	-9.76 ± 2.49	0.001**
ΔPLT	32.21 ± 4.50	39.34 ± 5.17	0.001**
ΔRBC	-0.42 ± 0.53	-0.60 ± 0.55	0.221
ΔHb	-17.72 ± 4.70	-18.4 ± 3.59	0.566
ΔESR	6.0 (2.0, 18.0)	3.0 (0.0, 12.0)	0.048*

Δ=0 weeks−32 week; independent sample t-tests and Mann-Whitney U test.

*P<0.05, **P<0.01, ***P<0.001.

### 3.4 Activity index evaluation

The CDAI scores of the two patient cohorts were analyzed for comparison. Initial assessments at baseline (0 week) indicated that the CDAI levels between the biologic-naïve and biologic-experienced groups were comparable, with no statistically significant difference observed (*P* > 0.05). However, after 32 week of treatment, the CDAI scores in the biologic-naïve group were significantly lower than those in the biologic-experienced group, demonstrating a statistically significant difference (*P* < 0.05). Further examination of the reduction in CDAI scores (ΔCDAI) revealed that the biologic-naïve group exhibited a greater decrease (136.25 ± 24.96) points compared to the biologic-experienced group (102.28 ± 24.00) points, suggesting a more pronounced trend in clinical improvement. This difference was also statistically significant (*P* < 0.05). Detailed data are presented in **Table 10**.

**Table 10:** Comparison of CDAI Scores between the two groups of patients.

Project	The biologic-naïve	The biologic-experienced	*P*
CDAI (0weeks)	254.56 ± 43.26	237.96 ± 59.15	0.68
CDAI (32weeks)	118.31 ± 38.30	135.68 ± 35.15	0.04*
ΔCDAI	136.25 ± 24.96	102.28 ± 24.00	0.03*

Δ=0 week−32 week; independent sample t-tests; *P<0.05, **P<0.01, ***P<0.001.

### 3.5 Endoscopic efficacy evaluation

The endoscopic efficacy between the two patient groups was assessed and compared. Initial analysis revealed that the baseline SES-CD levels at the commencement of treatment (week 0) were comparable between the biologic-naïve and biologic-experienced groups, with no statistically significant difference observed (*P* > 0.05). However, after 32 week of treatment, the SES-CD score for the biologic-naïve group was significantly lower than that of the biologic-experienced group, with the difference reaching statistical significance (*P* < 0.05). Further examination of the reduction in SES-CD values (Δ-SES-CD) demonstrated that the decrease in the biologic-naïve group (5.48 ± 0.21) points was greater than that observed in the biologic-experienced group (3.80 ± 0.57) points, suggesting a superior trend in endoscopic improvement for the biologic-naïve group. This difference was also statistically significant (*P* < 0.05). Detailed results are presented in **Table 11**.

**Table 11:** Comparison of SES-CD Scores between the two groups of patients.

Project	The biologic-naïve	The biologic-experienced	*P*
SES-CD (0weeks)	8.92 ± 1.79	9.11 ± 1.45	0.66
SES-CD (32weeks)	3.44 ± 1.58	5.31 ± 2.02	0.03*
ΔSES-CD	5.48 ± 0.21	3.80 ± 0.57	0.03*

Δ=0 week−32 week; independent sample t-tests; *P<0.05, **P<0.01, ***P<0.001.

### 3.6 Clinical outcomes

A comparative analysis was conducted on the clinical responses and clinical remission rates at the treatment endpoint (32 week) between the biologic-naïve group and the biologic-experienced group. Among the 21 patients in the biologic-naïve group, the clinical response rate was 61.9% (95% CI: 40.7% - 78.9%), and the clinical remission rate was 42.9% (95% CI: 23.9% - 63.7%). In contrast, the group of 40 patients with biologic-experienced exhibited a clinical response rate of 52.5% (95% CI: 37.9% - 66.8%) and a clinical remission rate of 40.0% (95% CI: 26.4% - 55.1%). The substantial overlap of the 95% confidence intervals for both clinical response and remission rates between the two groups indicates no significant difference in their short-term clinical efficacy, as detailed in **Table 12**.

**Table 12:** Comparison of clinical response and remission between biologic-naïve and biologic-experienced groups.

Groups	Clinical response n (%,95%CI)	Clinical remission n (%,95%CI)
The biologic-naïve	13 (61.9%,95%CI 40.7-78.9%)	9 (42.9%,95%CI 23.9-63.7%)
The biologic-experienced	21 (52.5%,95%CI 37.9-66.8%)	16 (40.0%, 95%CI 26.4-55.1%)

Categorical variables are presented as n (%). 95%CI was calculated using the Wilson score method.

### 3.7 IBDQ and HADS in the clinical response and non-response groups

#### 3.7.1 Comparison of IBDQ and HADS in the clinical response group

A comparative analysis was conducted on the quality of life and psychological status of patients in the clinical response group. The results showed that, compared to baseline values at week 0, there were improvements in quality of life, depression, and anxiety scores at week 32. Notably, quality of life significantly improved and the anxiety subscale (HADS-A) significantly decreased, with statistically significant differences(*P* < 0.05). However, no statistically significant difference was observed in the depression subscale (HADS-D)(*P >*0.05). See **Table 13** for details.

**Table 13:** Comparison of IBDQ and HADS in the clinical response group.

Project/time	0 week	32 week	*P*
IBDQ	157.45 ± 13.24	195.56 ± 10.22	0.001**
HADS-A	6.72 ± 3.69	5.44 ± 2.72	0.020*
HADS-D	5.16 ± 2.07	4.50 ± 1.85	0.051

Paired t-tests; *P<0.05 **P<0.01 ***P<0.001.

#### 3.7.2 Comparison of IBDQ and HADS in the clinical non-response group

A comparative analysis was conducted on the quality of life and psychological status of patients in the clinical non-response group. The results showed that, compared with the baseline (0 weeks), at 32 week of treatment, only the quality of life significantly improved, and the difference was statistically significant (*P* < 0.05). However, there were no statistically significant differences in the anxiety subscale (HADS-A) and depression subscale (HADS-D)(*P >*0.05), as detailed in **Table 14**.

**Table 14:** Comparison of IBDQ and HADS in the clinical non-response group.

Project/time	0 week	32 week	*P*
IBDQ	148.78 ± 15.54	169.34 ± 18.35	0.001**
HADS-A	8.39 ± 3.97	7.15 ± 3.24	0.073
HADS-D	6.32 ± 2.87	5.59 ± 2.75	0.156

Paired t-tests; *P<0.05 **P<0.01 ***P<0.001.

#### 3.7.3 Comparison of the changes in IBDQ and HADS between two groups

A comparative analysis was conducted on the improvements in quality of life, anxiety, and depression between the clinical response group and the clinical non-response group. The results showed that after 32 week of treatment, the improvement in IBDQ quality of life score in the clinical response group(-38.11 ± 3.02) points was significantly greater than that in the clinical non-response group (-20.56 ± 2.81) points (*P* < 0.05). However, there was no statistically significant difference in the improvement in anxiety (HADS-A) and depression (HADS-D) scores between the two groups (*P* > 0.05). Detailed results are presented in **Table 15**.

**Table 15:** Comparison of the changes in IBDQ and HADS scores between the two groups.

Project	The clinical response group	The clinical non-response group	*P*
ΔIBDQ	-38.11 ± 3.02	-20.56 ± 2.81	0.001**
ΔHADS-A	1.28 ± 0.97	1.24 ± 0.73	0.862
ΔHADS-D	0.66 ± 0.22	0.73 ± 0.12	0.246

Δ=0 weeks−32 week; independent sample t-tests; *P<0.05 **P<0.01 ***P<0.001.

## 4 Discussion

CD is a chronic inflammatory granulomatous condition predominantly affecting the terminal ileum and adjacent colon, though it can involve any segment of the gastrointestinal tract from the oral cavity to the anus, exhibiting a segmental distribution pattern. The primary clinical manifestations include abdominal pain, diarrhea, and weight loss. Additionally, CD may present with systemic symptoms such as fever and fatigue, alongside local complications such as perianal abscesses and fistulas, as well as extraintestinal manifestations affecting the joints, skin, and oral mucosa. The pathogenesis of CD remains poorly understood, but it is believed to result from the interplay of environmental, genetic, and intestinal microecological factors, culminating in an aberrant immune imbalance within the intestine. Therapeutic strategies encompass the management of inflammatory responses, symptomatic relief, and surgical interventions. In recent years, various biologic agents targeting the inflammatory pathways of CD have demonstrated significant therapeutic efficacy. Among these, UST is a monoclonal antibody that targets the IL-12/23 p40 subunit. By binding to this subunit, UST inhibits the biological activities of IL-12 and IL-23, thereby attenuating the associated inflammatory responses (15).

Multiple clinical studies have demonstrated that UST is effective in inducing and maintaining remission in patients with moderate to severe CD (16–18). Nonetheless, in clinical practice, further research is needed to fully understand the efficacy of UST as the biologic-naïve and as the biologic-experienced. Additionally, the effects of clinical response status on patients’ quality of life and psychological well-being remain under-explored. This study conducts a systematic retrospective analysis to evaluate the real-world clinical efficacy of UST, addressing these issues with the aim of providing comprehensive evidence-based insights to optimize clinical treatment strategies.

### 4.1 Dynamic changes and analysis of laboratory indicators after UST treatment

The results of this study indicate that over time, UST can improve the clinical symptoms and laboratory indicators of patients with CD as both the biologic-naïve and biologic-experienced option. However, there are certain differences in the improvement patterns between the two groups of patients, reflecting the characteristics of UST’s response in CD patients during the biologic-naïve and biologic-experienced groups. Laboratory indicator results show that for the biologic-naïve group: at the 8th week, all indicators showed an improvement trend compared to the baseline, but only the ESR level significantly decreased, with a statistically significant difference (*P* < 0.05). By the 32nd week, multiple laboratory indicators further improved compared to the 0th week. Among them, the CRP, PLT, and ESR levels significantly decreased, while the Hb level significantly increased. All differences were statistically significant (*P* < 0.05).

Analyzing the clinical phenomena in the 8th week and the 32nd week, the possible reasons are as follows: ① The observation period is relatively short. UST has a time lag effect on reversing the indicators related to chronic inflammation.The improvement of nutritional and anemia-related indicators such as ALB and Hb requires a longer recovery time (19). ② As the treatment time prolongs, intestinal inflammation is effectively controlled, manifested by significant decreases in inflammatory indicators such as CRP, ESR, and PLT (20). ③ After UST controls inflammation, the level of hepcidin decreases, iron metabolism disorder is corrected, and the hematopoietic function of the bone marrow is restored. At the same time, the intestinal mucosal injury is repaired, and the absorption functions of folic acid and vitamin B_12_ improve, thereby correcting the anemia of the patients and significantly increasing the hemoglobin level. Previous studies have shown that the PLT of patients with active CD is significantly higher than that of patients in remission, and PLT is closely related to the disease activity (21). After inflammation control, the platelet can decrease accordingly; while the improvement of nutritional absorption and correction of chronic anemia often manifest as an increase in ALB and Hb, which is consistent with the observed change pattern in this study.

Biologic-experienced group: At the 8th week of treatment, ESR showed significant improvement, suggesting that UST still has an early anti-inflammatory effect in patients who had failed previous biologic therapy (22). At the 32nd week of treatment, the ALB, PLT, RBC, HGB, and ESR in the biologic-experienced group all showed significant improvement (*P* < 0.05), indicating that UST can also improve the nutritional status and anemia of the second-line treatment group. There was no statistically significant improvement in CRP and WBC (*P* > 0.05). Possible reasons include: ① Patients in the biologic-experienced group had a history of previous biologic therapy failure. Previous biologic exposure may can affect the UST trough concentration, resulting in insignificant improvement in CRP (23). ② Some patients may have intestinal fibrosis or structural damage, and CRP levels may not fully reflect their active inflammatory burden. ③ Patients in the biologic-experienced group have a longer disease course and more severe immune dysfunction. It takes a longer time to observe a significant decrease in CRP. ④ The sample size is small or there are large individual differences, resulting in errors in the statistical results.

The baseline clinical data and laboratory indicators of the two groups of patients in this study were comparable. The changes in indicators before and after treatment were compared and analyzed between the two groups.The results showed that at 8 week, the differences in ΔCRP, ΔALB, ΔRBC, and ΔESR between the two groups were statistically significant (*P* < 0.05). The improvement in ALB in the experienced group was significantly greater than that in the naïve group, suggesting that the improvement in the nutritional status of the experienced patients was more prominent at an early stage. However, the reduction in CRP and ESR in the experienced group was weaker than that in the naïve group, indicating that even in the early stage of treatment, the decline of serum inflammatory markers in the experienced patients had certain limitations. At 32 week, the differences in ΔCRP, ΔALB, ΔPLT, and ΔESR were still statistically significant (*P* < 0.05); with the extension of treatment time, the differences in PLT changes were also statistically significant (*P* < 0.05). The decline in PLT in the experienced group was greater than that in the biologic-naïve group, while the decline in CRP and ESR was still less than that in the biologic-naïve group. These results suggest that the response patterns of UST to the two groups of patients are separated: the improvement in nutritional and anemia-related indicators in the biologic-experienced benefited more significantly, but the decline of serum CRP and ESR markers was less than that in the biologic-naïve group. At 8 week, the increase in RBC in the biologic-experienced group was better than that in the biologic-naïve group, but the difference between the two groups disappeared at 32 week, suggesting that the early improvement advantage of red blood cells in the treated group gradually narrowed with the extension of follow-up time. The changes in WBC and Hb at 8 week and 32 week showed no statistical differences between the two groups, indicating that the effects of UST in improving white blood cells and hemoglobin were similar in the biologic-naïve and biologic-experienced groups.

At 32 week, the biologic-naïve group showed significant improvements in inflammation and nutritional anemia indicators, indicating better control of inflammation and nutritional recovery. Although the biologic-experienced group also improved in several areas, their CRP levels did not significantly decrease, suggesting limited improvement in this marker. These patients had previously failed biological treatments and might have complex immune disorders or structural damage like intestinal fibrosis, affecting their drug response differently from the initial treatment group (24, 25). Thus, starting UST treatment early in CD patients may lead to more comprehensive inflammatory control.

### 4.2 Comparative analysis of clinical manifestations and endoscopic efficacy between the biologic-naïve and biologic-experienced groups

Further analysis of the reduction value (ΔCDAI) revealed that the reduction amplitude in the biologic-naïve group was greater than that in the biologic-experienced group. Possible reasons are as follows: ① The biologic-naïve group received UST intervention earlier, which enabled them to more promptly interrupt the inflammatory process and prevent irreversible structural damage to the intestine, thus achieving a more ideal disease control status at the end of the observation period; ② Patients in the biologic-experienced group had a history of treatment failure with other biological agents (such as anti-tumor necrosis factor drugs). Using one or more biological agents before UST would affect the body’s exposure to UST, thereby delaying the response ability of UST (26).

This study found that the ΔSES-CD score of the biologic-naïve group decreased more significantly, and the differences were also statistically significant (*P* < 0.05). The results of this study are consistent with the conclusions of Sedano et al.’s research (27). Their study found that under UST treatment, the endoscopic cure rate of patients receiving initial biological therapy was significantly higher than that of patients previously receiving biological therapy, and the median time to achieve endoscopic remission was shorter (8.4 months vs 15.4 months). Possible reasons include: Exposure history may be a key factor affecting the treatment outcome. Patients in the biologic-naïve group had not received biological therapy before, and their inflammatory pathways may not have undergone complex adaptive changes, so they were more sensitive to the blockade of IL-12/23 (28).

The study further compared clinical response and remission rates between two patient groups. The biologic-naïve group had rates of 61.9% and 42.9%, while the biologic-experienced group had rates of 52.5% and 40.0%.This is consistent with the findings of another study, which indicated that for patients newly treated with biological agents, their clinical responses and remission rates were higher than those of patients who had received treatment with biological agents before (29). Despite higher rates in the biologic-naïve group, overlapping 95% confidence intervals suggest no statistical difference between the groups. This indicates that UST provides significant clinical benefits for CD patients, even those who previously failed biologic treatments.It is important to acknowledge that the findings of this study do not completely correspond with the observed inter-group variations in serological indicators. Specifically, while the biologic-experienced group exhibited only modest improvements in systemic inflammatory markers such as C-reactive protein (CRP), a significant proportion of patients nonetheless achieved clinical responses and remissions. This suggests a potential dissociation between peripheral blood inflammatory markers and the amelioration of clinical symptoms (30). However, the sample size of this study is limited, and these findings still need to be verified through larger-scale research.

### 4.3 Relationship between clinical response and quality of life

At 32 week, the total scores of IBDQ in both the clinical response group and the non-response group significantly increased compared to the baseline (*P* < 0.01), indicating that regardless of whether a clinical response was achieved, the treatment could improve the overall quality of life of patients. However, the increase in the total score of IBDQ in the response group was significantly greater than that in the non-response group (*P* < 0.01), suggesting that a clinical response may be an important condition for patients to benefit more from their quality of life (31). The difference between the two groups originated from the different treatment outcomes of the two groups: the intestinal inflammation was effectively controlled in the response group, while the disease continued to be active in the non-response group. Disease activity could affect the overall quality of life through multiple aspects such as intestinal symptoms, negative emotions, and impaired social function (32–34).At the same time, systemic symptoms such as fatigue are regulated by multiple factors including inflammation, nutrition, and psychology, and may not fully recover even when inflammation is relieved, which will to some extent limit the improvement of quality of life (35). Combined with the ΔHADS results, there was no statistically significant difference in the change range of anxiety and depression scores between the two groups, suggesting that the inter-group difference in quality of life improvement cannot be simply explained by emotional changes, and intestinal symptom control remains the key to determining the overall improvement of IBDQ (36).

This study used the HADS to assess the psychological state of patients at 32 week. The results showed that the clinical response group had significantly lower HADS-A anxiety scores than the non-response group; although the HADS-D depression scores of the response group showed a lower trend, there was no statistically significant difference between the groups (*P >*0.05). The non-response group continued to have intestinal inflammation, which was influenced by the neuro-immune pathway in the central nervous system, inducing anxiety, and forming a vicious cycle of inflammation-anxiety through the brain-gut axis (37); the social avoidance caused by intestinal symptoms further exacerbated the psychological burden (38). The symptoms of the response group were controlled, and social life was not significantly restricted, and the anxiety level was relatively lower. Both groups did not reach the depression screening threshold for HADS-D, but there were still some patients with prominent depressive symptoms within the groups. Previous studies have shown that the risk of depression in IBD patients is higher than that in the general population, although it is related to disease activity, it is also influenced by multiple factors such as genetics and social psychology, and does not completely alleviate with inflammation control (39–41). There is a time lag in the improvement of depression, and a 32-week follow-up may not be sufficient to observe the significant differentiation of depression levels between the two groups.

In conclusion, the differences in quality of life between the clinical response group and the non-response group stem from the different treatment outcomes. The clinical response group achieved effective control of disease activity after treatment, with a significant reduction in intestinal symptom burden, leading to simultaneous improvements in emotional and social functions; while the non-response group experienced continuous disease activity and persistent physical symptoms, which resulted in impaired emotional state and social participation ability. There was no significant difference in the dimension of systemic symptoms between the two groups, suggesting that the improvement of fatigue and other systemic symptoms may lag behind the relief of intestinal inflammation. It is regulated by multiple non-inflammatory factors and even achieving clinical response may require a longer time for the recovery of such symptoms. Considering the results of psychological status, patients who achieved clinical response had significantly lower anxiety levels than those in the non-response group, and this benefit was closely related to disease control and improvement in quality of life. There was no statistical difference in depression between the two groups, and depression is influenced by multiple factors such as genetics and social psychology, not solely determined by disease activity. The 32-week follow-up period was not sufficient to observe significant differentiation in depression levels between the two groups. These results suggest that achieving clinical response is an important prerequisite for improving the quality of life and anxiety of patients with CD. For patients with comorbid depression, longer follow-up observation is required, and comprehensive intervention measures may be necessary. Psychological assessment should be included in the long-term follow-up system to implement the integrated management model of physical and mental treatment.

### 4.4 Analysis of limitations

This study has certain limitations. As a single-center retrospective study, it is subject to selection bias with limited generalizability. The small overall sample and unbalanced group sizes may reduce statistical power. Heterogeneous treatment indications and unadjusted potential confounders may influence outcome interpretation. Besides, only partial patients completed week-32 endoscopy, so categorical endoscopic endpoints could not be evaluated for the whole cohort. Fecal calprotectin and therapeutic drug monitoring data were also absent. Due to the limitation of sample size, this study also cannot conduct further stratified analysis to explore the association between the baseline Montreal classification phenotype and clinical treatment response. Further multi-center prospective studies with larger sample sizes are needed to confirm our findings.

## 5 Conclusion

UST can improve the inflammation and nutritional status of patients with CD who are biologic-naïve or biologic-experienced. Compared to those with prior biologic experience, biologic-naive patients may have certain advantages in terms of inflammation control and recovery of nutritional parameters. Early initiation of UST therapy in CD patients might lead to better inflammation control. Moreover, clinical response is closely associated with a comprehensive improvement in quality of life. Anxiety symptoms can be somewhat alleviated, while the depressive state has not shown any significant change.

## Data Availability

The raw data supporting the conclusions of this article will be made available by the authors, without undue reservation.
