# Supplementary material for: Retrospective analysis of ustekinumab in Crohn’s disease between biologic-naïve and biologic-experienced
Source: Front Gastroenterol (Lausanne). 2026 Sep 22;5:1926425. doi: 10.3389/fgstr.2026.1926425 (PMC13638564; doi:10.3389/fgstr.2026.1926425)
Supplement: Supplementary file 1 [file Table1.docx]

Appendix 1 Montreal Classification Criteria for Crohn's Disease

| **Classification dimension** | **Classification criteria** |
| --- | --- |
| **Diagnosis age (A)** |  |
| A1 | ≤16 |
| A2 | 17-40 |
| A3 | >40 |
| **Site of onset (L)** |  |
| L1 | Terminal ileum |
| L2 | Colon |
| L3 | ileocolon |
| L4 | upper gastrointestinal tract |
| **Disease Behavior (B)** |  |
| B1 | Non-constrictive non-penetrating type |
| B2 | Narrow type |
| B3 | Penetrating type |
| P | Perianal lesion |

Note: B4 can coexist with L1, L2, and L3. As the disease progresses, B1 can develop into B2 or B3. Perianal lesions (P) can coexist with B1, B2, and B3.

Appendix 2 Crohn's Disease Activity Index (CDAI) Score

| **Variable** | **Weight** |
| --- | --- |
| Frequency of loose stools (1 week) | 2 |
| Degree of abdominal pain (1-week total score, 0-3 points) | 5 |
| General condition (1-week total score, 0-4 points) | 7 |
| Extraintestinal manifestations and complications* (1 point for each item) | 20 |
| Use of opioid antidiarrheal drugs (0, 1 point) | 30 |
| Abdominal mass (0 points for no mass, 2 points for suspected, 5 points for definite) | 10 |
| Decrease in hematocrit value (normal: 0.40 for men, 0.37 for women) | 6 |
| 100 × (1 - weight / standard weight) | 1 |

Note: a CDAI score of ≥150 points was defined as clinical active phase (where 150 ≤ CDAI ≤ 220 indicated mild activity, 221 < CDAI ≤ 450 indicated moderate activity, and CDAI > 451 indicated severe activity);Extra-intestinal manifestations and complications include arthritis/joint pain, iritis/iritis, erythema nodosum/UC/oral ulcers, anal fissure/anorectal fistula/anorectal abscess, other intestinal-related fistulas, fever (body temperature exceeding 38℃ within 1 week).

**Appendix 3: Inflammatory Bowel Disease Questionnaire (IBDQ)**

Hello, this is a questionnaire about quality of life in inflammatory bowel disease. Please select the option that best reflects your actual feelings and condition over the past two weeks. Your responses will be strictly confidential. Thank you for your support and cooperation.

1. I have abdominal pain ( )

A. Severely affected B. Very severely affected C. Moderately affected

D. Slightly affected E. Almost unaffected F. Not affected G. Completely unaffected

2. I have diarrhea( )

A. Severely affected B. Very severely affected C. Moderately affected

D. Slightly affected E. Almost unaffected F. Not affected G. Completely unaffected

3. I have bloody stool ( )

A. Severely affected B. Very severely affected C. Moderately affected

D. Slightly affected E. Almost unaffected F. Not affected G. Completely unaffected

4. I have abdominal distension ( )

A. Severely affected B. Very severely affected C. Moderately affected

D. Slightly affected E. Almost unaffected F. Not affected G. Completely unaffected

5. I have a sense of urgency for defecation ( )

A. Severely affected B. Very severely affected C. Moderately affected

D. Slightly affected E. Almost unaffected F. Not affected G. Completely unaffected

6. Feeling of incomplete defecation ()

A. Severely affected B. Very severely affected C. Moderately affected

D. Slightly affected E. Almost unaffected F. Not affected G. Completely unaffected

7. I need to get up at night to have a bowel movement ( )

A. Severely affected B. Very severely affected C. Moderately affected

D. Slightly affected E. Almost unaffected F. Not affected G. Completely unaffected

8. My gastrointestinal symptoms have affected my social activities. ( )

A. Severely affected B. Very severely affected C. Moderately affected

D. Slightly affected E. Almost unaffected F. Not affected G. Completely unaffected

9. My gastrointestinal symptoms have affected my work/study ( )

A. Severely affected B. Very severely affected C. Moderately affected

D. Slightly affected E. Almost unaffected F. Not affected G. Completely unaffected

10. I'm worried that the intestinal symptoms might suddenly flare up ( )

A. Severely affected B. Very severely affected C. Moderately affected

D. Slightly affected E. Almost unaffected F. Not affected G. Completely unaffected

11. I feel tired/weak ( )

A. Severely affected B. Very severely affected C. Moderately affected

D. Slightly affected E. Almost unaffected F. Not affected G. Completely unaffected

12. My appetite has decreased ( )

A. Severely affected B. Very severely affected C. Moderately affected

D. Slightly affected E. Almost unaffected F. Not affected G. Completely unaffected

13. I have poor sleep quality ( )

A. Severely affected B. Very severely affected C. Moderately affected

D. Slightly affected E. Almost unaffected F. Not affected G. Completely unaffected

14. I feel exhausted ( )

A. Severely affected B. Very severely affected C. Moderately affected

D. Slightly affected E. Almost unaffected F. Not affected G. Completely unaffected

15. I have a fever or night sweats ( )

A. Severely affected B. Very severely affected C. Moderately affected

D. Slightly affected E. Almost unaffected F. Not affected G. Completely unaffected

16. I feel anxious/tense ( )

A. Severely affected B. Very severely affected C. Moderately affected

D. Slightly affected E. Almost unaffected F. Not affected G. Completely unaffected

17. I feel depressed/low in spirits ( )

A. Severely affected B. Very severely affected C. Moderately affected

D. Slightly affected E. Almost unaffected F. Not affected G. Completely unaffected

18. I am not satisfied with my body image ( )

A. Severely affected B. Very severely affected C. Moderately affected

D. Slightly affected E. Almost unaffected F. Not affected G. Completely unaffected

19. I'm worried that the illness will affect my future ( )

A. Severely affected B. Very severely affected C. Moderately affected

D. Slightly affected E. Almost unaffected F. Not affected G. Completely unaffected

20. I feel irritable/ have a lot of mood swings ( )

A. Severely affected B. Very severely affected C. Moderately affected

D. Slightly affected E. Almost unaffected F. Not affected G. Completely unaffected

21. I feel ashamed of my illness ( )

A. Severely affected B. Very severely affected C. Moderately affected

D. Slightly affected E. Almost unaffected F. Not affected G. Completely unaffected

22. I feel lonely/isolated ( )

A. Severely affected B. Very severely affected C. Moderately affected

D. Slightly affected E. Almost unaffected F. Not affected G. Completely unaffected

23. I'm worried that illness might affect my close relationships ( )

A. Severely affected B. Very severely affected C. Moderately affected

D. Slightly affected E. Almost unaffected F. Not affected G. Completely unaffected

24. I am worried about the treatment outcome ( )

A. Severely affected B. Very severely affected C. Moderately affected

D. Slightly affected E. Almost unaffected F. Not affected G. Completely unaffected

25. I feel helpless/desperate ( )

A. Severely affected B. Very severely affected C. Moderately affected

D. Slightly affected E. Almost unaffected F. Not affected G. Completely unaffected

26. I am frustrated about my health condition ( )

A. Severely affected B. Very severely affected C. Moderately affected

D. Slightly affected E. Almost unaffected F. Not affected G. Completely unaffected

27. I'm worried that the illness might affect my fertility ( )

A. Severely affected B. Very severely affected C. Moderately affected

D. Slightly affected E. Almost unaffected F. Not affected G. Completely unaffected

28. My illness has affected my ability to work/study ( )

A. Severely affected B. Very severely affected C. Moderately affected

D. Slightly affected E. Almost unaffected F. Not affected G. Completely unaffected

29. My illness has restricted my social activities ( )

A. Severely affected B. Very severely affected C. Moderately affected

D. Slightly affected E. Almost unaffected F. Not affected G. Completely unaffected

30. My illness has affected my relationship with my family ( )

A. Severely affected B. Very severely affected C. Moderately affected

D. Slightly affected E. Almost unaffected F. Not affected G. Completely unaffected

31. My illness has restricted my leisure and recreational activities ( )

A. Severely affected B. Very severely affected C. Moderately affected

D. Slightly affected E. Almost unaffected F. Not affected G. Completely unaffected

32. I need to rely on others to carry out daily activities ( )

A. Severely affected B. Very severely affected C. Moderately affected

D. Slightly affected E. Almost unaffected F. Not affected G. Completely unaffected

Note: The Inflammatory Bowel Disease Quality of Life Questionnaire (IBDQ) is a specific scale specifically designed to assess the quality of life of patients with inflammatory bowel disease. It consists of 4 dimensions: intestinal symptoms, systemic symptoms, emotional function, and social function, with a total of 32 items. Each item is scored on a scale of 1 to 7. The total score is obtained by simply adding up all the individual scores. The total score ranges from 32 to 224. A higher total score indicates better quality of life for the patient.

**Appendix 4: Hospital Anxiety and Depression Scale (HADS)**

Hello, this is a self-assessment questionnaire about your recent emotional state. Please choose the option that best reflects your feelings over the past week. Each question can only have one answer. Your responses will be kept strictly confidential. Thank you for your support and cooperation.

HADS-A

1. I feel nervous (or painful):

□ Almost never (0)	□ Sometimes (1)	□ Most of the time (2)	□ Almost always (3)

2. I felt a little scared, as if something terrible was about to happen:

□ Almost never (0)	□ Sometimes (1)	□ Most of the time (2)	□ Almost always (3)

3. My heart is filled with worries:

□ Almost never (0)	□ Sometimes (1)	□ Most of the time (2)	□ Almost always (3)

4. I felt a bit restless and couldn't sit still; I seemed to need to move around.

□ Almost never (0)	□ Sometimes (1)	□ Most of the time (2)	□ Almost always (3)

5. I feel confused or frightened:

□ Almost never (0)	□ Sometimes (1)	□ Most of the time (2)	□ Almost always (3)

6. I suddenly felt a sense of panic:

□ Almost never (0)	□ Sometimes (1)	□ Most of the time (2)	□ Almost always (3)

7. I can sit comfortably and feel relaxed:

□ Almost never (0)	□ Sometimes (1)	□ Most of the time (2)	□ Almost always (3)

HADS-D

1. I can laugh and see the positive side of things:

□ Almost never (0)	□ Sometimes (1)	□ Most of the time (2)	□ Almost always (3)

2. It seems that my mood is gradually sinking.

□ Almost never (0)	□ Sometimes (1)	□ Most of the time (2)	□ Almost always (3)

3. I feel relieved.

□ Almost never (0)	□ Sometimes (1)	□ Most of the time (2)	□ Almost always (3)

4. My interest in things has waned:

□ Almost never (0)	□ Sometimes (1)	□ Most of the time (2)	□ Almost always (3)

5. I think life is very meaningful:

□ Almost never (0)	□ Sometimes (1)	□ Most of the time (2)	□ Almost always (3)

6. I feel that everything is of no value:

□ Almost never (0)	□ Sometimes (1)	□ Most of the time (2)	□ Almost always (3)

7. I can appreciate a good book or a good program:

□ Almost never (0)	□ Sometimes (1)	□ Most of the time (2)	□ Almost always (3)

Note: The Hospital Anxiety and Depression Scale (HADS) is an emotional screening scale specifically designed for patients with physical illnesses. It does not include items related to physical symptoms and is applicable to patients with inflammatory bowel disease. The Anxiety Subscale (HADS-A) assesses anxiety, while the Depression Subscale (HADS-D) assesses depression. The higher the score, the more obvious the corresponding emotional symptoms. This study uses the commonly used cutoff values: a score of ≥8 for the subscale indicates the presence of anxiety/depression, and a score of <8 indicates no significant anxiety/depression.
